# Liquid Chromatographic Determination of Linagliptin in Bulk, in Plasma and in its Pharmaceutical Preparation

**Published:** 2012-09

**Authors:** Ramzia I. El-Bagary, Ehab F. Elkady, Bassam M. Ayoub

**Affiliations:** *Department of Pharmaceutical Chemistry, Faculty of Pharmacy, Cairo University, Kasr El-Aini St., Cairo 11562, Egypt*

**Keywords:** linagliptin, reversed-phase liquid chromatography, fluorometric detection, pharmaceutical preparation, plasma

## Abstract

In this work, two reversed-phase liquid chromatographic (RP-LC) methods have been developed for the determination of linagliptin (LNG) based on isocratic elution using a mobile phase consisting of potassium dihydrogen phosphate buffer pH (4.6)-acetonitrile(20:80, *v/v*) at a flow rate of 1 mL min^−1^. Two detection techniques have been applied either UV detection at 299 nm in the first method or fluorometric detection at 239 nm for excitation and 355 nm for emission in the second method. Chromatographic separation in the two methods was achieved on a Symmetry^®^ cyanide column (150 mm × 4.6 mm, 5 μm). Linearity, accuracy and precision were found to be acceptable over the concentration ranges of 2.5-80 μg mL^−1^ for LNG in bulk and 2.5-15 μg mL^−1^ for LNG in plasma with the first method and 5-160 μg mL^−1^ for LNG in bulk with the second method. The optimized methods were validated and proved to be specific, robust and accurate for the quality control of the cited drug in its pharmaceutical preparation.

## INTRODUCTION

**Figure 1:**
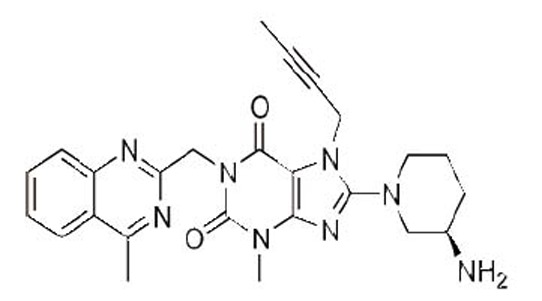
Chemical structure of linagliptin.

Linagliptin (LNG), 8-[(3R)-3-aminopiperidin-1-yl]-7-(but-2-yn-1-yl)-3- methyl-1-[(4-methylquinazolin-2-yl)methyl]-3,7-dihydro-1H-purine-2,6-dione] (Fig. 1) is a novel hypoglycemic drug that belongs to dipeptidyl-peptidase-4 inhibitor class (1, 2). DPP-4 inhibitors represent a new therapeutic approach to the treatment of type 2 diabetes that functions to stimulate glucose-dependent insulin release and reduce glucagon levels. This is done through inhibition of the inactivation of incretins, particularly glucagon-like peptide-1 (GLP-1) and gastric inhibitory polypeptide (GIP), thereby improving glycemic control (3). Recently, DPP-4 inhibitors have been recommended in the treatment of diabetes mellitus to improve glycemic control (4) and it is effective in controlling the metabolic syndrome and resulted in significant weight loss, a reversal of insulin resistance, islet and adipocyte hypertrophy, and alleviated hepatic steatosis (5).

Only one method has been described for the determination of LNG in its pharmaceutical preparation based on reversed-phase liquid chromatography (6).

Due to the native fluorescence of LNG, our aim was to compare the two techniques of detection widely applied in routine analysis; namely UV and fluorometric detection and to try to develop a more sensitive method than that reported. Thus, we developed alternative LC methods for the determination of LNG and applied it to the determination of LNG in plasma. In the first method (LC-UV), UV detection was applied for the determination of LNG in bulk, in plasma and in its dosage form. In the second method (LC-fluoro), LNG was determined in bulk and in its dosage form applying fluorometric detection based on the native fluorescence of the drug.

## EXPERIMENTAL

### Instrumentation

The HPLC system consisted of a Schimadzu LC-20 AT Liquid Chromatograph (Japan) using a Symmetry^®^ cyanide column (150 mm × 4.6 mm, 5 μm). The system was equipped with a flourometric detector (RF-551, Japan), UV-visible detector (SPD-20A, Japan) and an autosampler (SIL-20A, Schimadzu, Japan). An Elma S100 ultrasonic processor model KBK 4200 (Germany) was used.

### Reagents and reference samples

Pharmaceutical grade LNG, certified to contain 99.80%, Tradjenta^®^ tablets nominally containing 5 mg of LNG per tablet were supplied from Eli Lilly and company (USA). HPLC grade acetonitrile and methanol were purchased from Fisher Scientific (Loughborough, Leicestershire, UK). Potassium dihydrogen phosphate and orthophosphric acid (85%) were purchased from VWR Chemicals (Pool, England). Bi-distilled water was produced in-house (Aquatron Water Still, A4000D, UK). Membrane filters 0.45 μm from Teknokroma (Barcelona, Spain) were used. All other chemicals and reagents used were of analytical grade unless indicated otherwise. Standard stock solutions of LNG (1 mg mL^-1^) were prepared by dissolving 100 mg of LNG in methanol in a 100 mL volumetric flask and completing to volume with methanol. The required concentrations were prepared by serial dilutions.

### Plasma sample preparation

The spiked plasma samples of LNG were extracted after precipitation of proteins using 100 μL of perchloric acid (35% *w/w*). Then, the mixture was vortex-mixed and centrifuged (3 min). The supernatant was separated and transferred to another tube and a 25 μl volume was injected into the chromatograph.

### Chromatographic conditions

Chromatographic separation was achieved on a Symmetry^®^ cyanide column (150 mm × 4.6 mm, 5 μm) applying an isocratic elution based on potassium dihydrogen phosphate buffer pH (4.6) - acetonitrile (20:80, *v/v*) as a mobile phase. The buffer solution was filtered through 0.45 μm membrane filter and degassed for 30 min in an ultrasonic bath prior to its use. The mobile phase was pumped through the column at a flow rate of 1 mL min^-1^. For LC-UV method, the UV detector was operated at 299 nm. For LC-fluoro method, the fluorometric detector was operated at 239 nm for excitation and 355 nm for emission. Analyses were performed at ambient temperature and the injection volume was 25 μL.

### Sample preparation

Twenty tablets of Tradjenta^®^ were weighed. An accurately weighed amount of the finely powdered Tradjenta^®^ tablets equivalent to 100 mg of LNG were separately made up to 100 mL with methanol and sonicated to dissolve. The solutions were filtered followed by serial dilutions to the required concentrations for each experiment.

### Procedure

**Linearity and repeatability.**

- **LC-UV method in bulk.** Accurately measured aliquots of stock solutions equivalent to 25-800 µg LNG were transferred into two series of 10 mL volumetric flasks and then completed to volume with methanol. A volume of 25 µL of each solution was injected into the chromatograph. The conditions including the mobile phase at a flow rate 1 mL min^-1^ and detection at 299 nm were adjusted. A calibration curve for LNG was obtained by plotting area under the peak (AUP) against concentration (C). The repeatability of the method was assessed by analyzing 50 µg mL^-1^ of LNG (*n*=6). The precision (%R.S.D) values of peak areas and retention times were calculated for each compound, Table 1.
- **LC-UV method in plasma.** Accurately measured aliquots of plasma samples equivalent to 2.5-15 μg LNG were prepared after its extraction as mentioned under **Plasma sample preparation.** with the same conditions under **LC-UV method in bulk.** A calibration curve was obtained by plotting area under the peak (AUP) against concentration (C). The repeatability of the method was assessed by analyzing 10 μg mL^-1^ of LNG, respectively (*n*=6). The precision (%R.S.D) was calculated, Table 1.
- **LC-fluoro method.** Accurately measured aliquots of LNG stock solution equivalent to 50-1600 μg were transferred into a series of 10 mL volumetric flasks and then completed to volume with methanol. A volume of 25 μL of each solution was injected into the chromatograph. The conditions including the mobile phase at a flow rate 1 mL min^-1^ and fluorometric detection (λ_ex_ 239 nm, λ_em_ 355 nm) were adjusted. A calibration curve was obtained by plotting area under the peak (AUP) against concentration(C). The repeatability of the method was assessed by analyzing 50 μg mL^-1^ of LNG (*n*=6).The precision (%R.S.D) values of peak areas and retention times were calculated, Table 1.

**Assay of LNG in bulk, plasma and Tradjenta^®^ tablets.** The procedure mentioned under **LC-UV method in bulk** was repeated using concentrations equivalent to 15-75 µg mL^-1^ LNG in bulk and equivalent to 4-12 µg mL^-1^ in plasma samples. The procedure mentioned under 2.6.1.2. was repeated using concentrations equivalent to 15-135 µg mL^-1^ LNG in bulk. For the determination of LNG in Tradjenta^®^ tablets, the sample solution prepared under 2.6. was serially diluted and then injected in triplicates. The concentrations of LNG were calculated using calibration equation of each method.

## RESULTS AND DISCUSSION

HPLC greatly reduces the analysis time and allows for the determination of many individual components in a mixture using one single procedure (7). Due to the native fluorescence of LNG, we studied the two techniques of detection widely applied in routine analysis; namely UV and fluorometric detection. UV detection was selected and applied for the determination of LNG in plasma due to its more sensitivity and its applicability to the lower concentrations of LNG without distortions in the peak, also it gives linear reproducible results as shown in Table 2.

### Methods development

**Figure 2:**
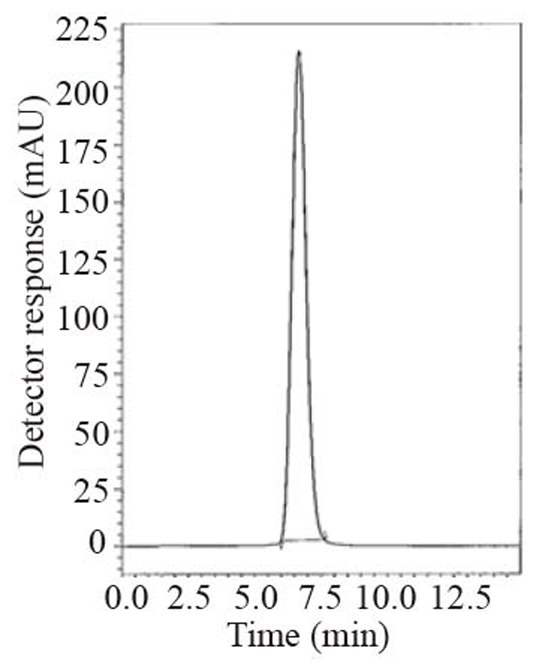
A typical LC chromatogram with ultraviolet detection of 25 μL injector of Tradjenta^®^ sample solution (50 μg mL^-1^).

**Figure 3:**
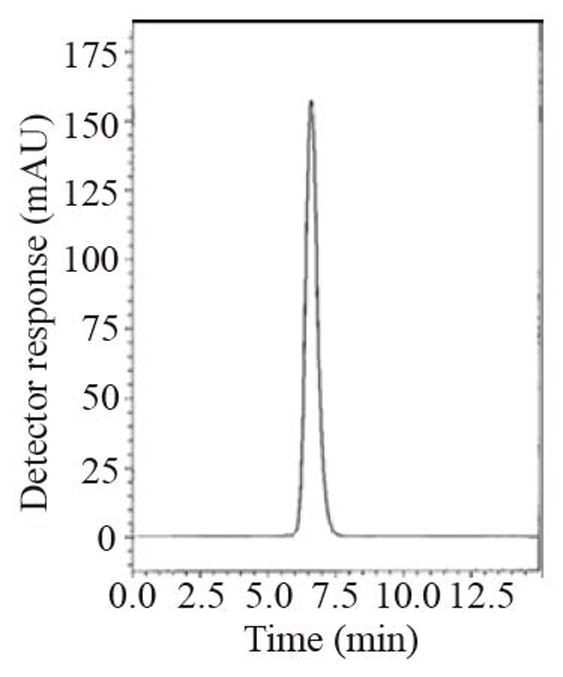
A typical LC chromatogram with ultraviolet detection of 25 μL injector of linagliptin in plasma (10 μg mL^-1^).

During the optimization cycle, various reversed-phase columns, isocratic mobile phase systems and different pH values of the buffer were attempted. Symmetry^®^ cyanide column (150 mm × 4.6 mm, 5 μm) was found optimum. Various mobile phase compositions containing different ratios of organic and aqueous phases were tried in an isocratic mode. Acetonitrile was found optimum for the elution. Besides, different buffers at different pH values were attempted along with acetonitrile. Therefore, a mobile phase consisting of potassium dihydrogen phosphate buffer pH (4.6) - acetonitrile (20:80, *v/v*) pumped at a flow rate of 1.0 mL min^-1^, in an isocratic mode, gave good result. In LC-UV method, detection was carried out at 299 nm. In LC-Fluoro method, the fluorometric detector was operated at 239 nm for excitation and 355 nm for emission where high detector sensitivity was achieved at these wavelengths. The retention time was 6.6 min for LNG as in Fig. 2 and the retention time was 6.5 min for LNG in plasma as shown in Fig. 3.

### System suitability tests

According to USP 2007 (8), system suitability tests are an integral part of liquid chromatographic methods in the course of optimizing the conditions of the proposed method. System suitability tests are used to verify that the resolution and reproducibility were adequate for the analysis performed. The parameters of these tests are column efficiency (number of theoretical plates), tailing of chromatographic peak and repeatability as %R.S.D of peak area for six injections and reproducibility of retention as %R.S.D of retention time. The results of these tests for the two proposed methods are listed in Table 1.

**Table 1:** System suitability tests for LC-UV method for the determination of linagliptin in bulk and in plasma and for LC-fluoro method for the determination of linagliptin in bulk

Item	LC-UV methodin bulk	LC-UV methodin plasma	LC-fluoro method

N	957	1076	842
T	1.04	1.03	1.07
RSD% of 6 injections			
Peak area	0.21	0.57	0.22
Retention time	0.34	0.46	0.69

### Methods validation

**Linearity.**

- **LC-UV method.** A linear relationship between area under the peak (AUP) and components’ concentrations (C) was obtained. The regression equation was computed, Table 2 for the method in bulk and in plasma. The linearity of the calibration curves were validated by the high value of correlation coefficients. The analytical data of the calibration curves including standard deviations for the slope and intercept (S_b_, S_a_) are summarized in Table 2.
- **LC-fluoro method.** A linear relationship between area under the peak (AUP) and LNG concentration (C) was obtained. The regression equation was computed, Table 2. The linearity of the calibration curve was validated by the high value of correlation coefficient. The analytical data of the calibration curve including standard deviations for the slope and intercept (S_b_, S_a_) are summarized in Table 2.

**Table 2:** Results obtained for LC-UV method for the determination of linagliptin in bulk and in plasma and for LC-fluoro method for the determination of linagliptin in bulk

Item	UV method in bulk	UV method in plasma	Fluoro method

Retention time	6.6	6.5	5.5
Wavelength of detection	299 nm	299 nm	239 nm for excitation
			355 nm for emission
Range of linearity	2.5-80 μg.ml^-1^	2.5-15 μg.ml^-1^	5-160 μg.ml^-1^
Regression equation	Area × 10^-5^ = 0.4966 C_μg/ml_ - 0.2808	Area × 10^-4^ = 2.0931 C_μg/ml_ - 0.6823	Area × 10^-5^ = 1.0095 C_μg/ml_ - 0.5191
Regression coefficient (r^2^)	0.9999	0.9987	0.9999
LOD μg.ml^-1^	0.73	0.56	1.29
LOQ μg.ml^-1^	2.44	1.87	4.28
S_b_	1.8 × 10^-3^	1.7 × 10^-2^	1.6 × 10^-3^
S_a_	0.08	0.21	0.15
Confidence limit of the slope	0.4966± 0.04	2.0931 ± 0.44	1.0095 ± 0.15
Confidence limit of the intercept	-0.2808± 0.51 × 10^-3^	-0.6823 ± 1.16× 10^-2^	-0.5191 ± 0.83× 10^-3^
Standard error of the estimation	0.12	0.18	0.21
Intraday %R.S.D	0.19-0.62	0.41-0.84	0.38-0.67
Interday %R.S.D	0.21-1.22	0.33-0.80	0.66-1.11
Drug inbulk	99.98 ± 1.13	100.54 ± 0.83	100.20 ± 1.27
Drug in dosage form	99.95 ± 0.99		100.12 ± 1.64
Drug added	99.90 ± 1.16		99.72 ± 1.59

**Accuracy.**

- **LC-UV method.** Accuracy of the results was calculated by % recovery of 5 different concentrations of LNG and also by standard addition technique applied for Tradjenta^®^ tablets, all carried out in triplicates. The results obtained including the mean of the recovery and standard deviation are displayed in Table 2.
- **LC-fluoro method.** Accuracy of the results was calculated by % recovery of 5 different concentrations of LNG and also by standard addition technique applied for Tradjenta^®^ tablets, all carried out in triplicates. The results obtained including the mean of the recovery and standard deviation are displayed in Table 2.

**Precision.**

- **LC-UV method.** The repeatability of the method was assessed by six determinations for each of the three concentrations of the laboratory prepared mixture of LNG (40-50-60 μg.ml^-1^) representing 80-100-120%, respectively. The repeatability of sample application and measurement of peak area for active compound were expressed in terms of percentage relative standard deviation (%R.S.D.) and found to be less than 1% in the three concentrations. Intra-day and inter-day precision (using 3 different concentrations in triplicates for three consecutive days) for LNG and in bulk and in plasma was also calculated. Results for the determination of precision are displayed in Tables 1, 2.
- **LC-fluoro method.** The repeatability of the method was assessed by six determinations for each of the three concentrations of the laboratory prepared mixture of LNG (40-50-60 μg.ml^-1^) representing 80-100-120%, respectively. The repeatability of sample application and measurement of peak area for active compound were expressed in terms of percentage relative standard deviation (%R.S.D.) and found to be less than 1% in the three concentrations. Results for the determination of precision are displayed in Tables 1, 2.

### Specificity

**Figure 4:**
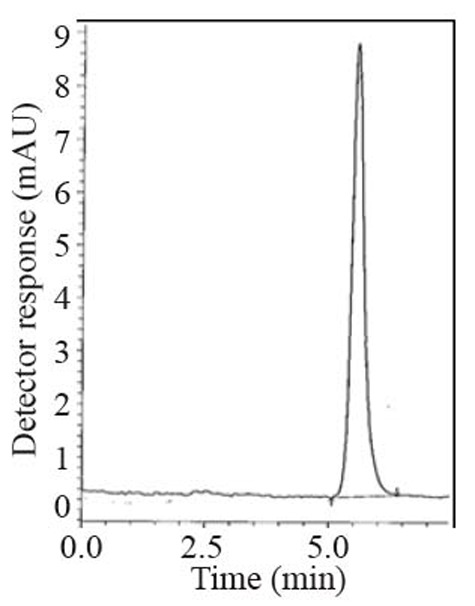
A typical LC chromatogram with fluorometric detection of 25 μL injector of Tradjenta^®^ sample solution (50 μg mL^-1^).

Specificity is the ability of the analytical method to measure the analyte response in the presence of interferences including degradation products and related substances. The chromatograms of the samples were checked for the appearance of any extra peaks. No chromatographic interference from any of the excipients was found at the retention times of the examined compounds (Fig. 2-4). In addition, the chromatogram of each compound in the sample solution was found identical to the chromatogram received by the standard solution at the wavelengths applied. These results demonstrate the absence of interference from other materials in the pharmaceutical formulations and therefore confirm the specificity of the proposed methods.

### Limit of detection and limit of quantification

Limit of detection (LOD) which represents the concentration of analyte at S/N ratio of 3 and limit of quantification (LOQ) at which S/N is 10 were determined experimentally for the proposed methods and results are given in Table 2.

### Statistical analysis

A statistical analysis of the results obtained by the proposed method and the reference method was carried out by “SPSS statistical package version 11”. The significant difference between groups was tested by one way ANOVA (F-test) at *p*=0.05 as shown in Table 3. The test ascertained that there was no significant difference among the methods.

**Table 3:** Statistical comparison between the results of proposed methods and the reference method for the determination of linagliptin

StatisticalTerm	Reference Method^b^	LC-UV	LC-UV plasma	LC-fluoro

Mean	99.45	99.98	100.54	100.20
S.D.±	1.34	1.13	0.83	1.27
S.E. ±	0.60	0.51	0.37	0.57
%RSD	1.35	1.13	0.83	1.27
n	5	5	5	5
V	1.80	1.28	0.69	1.61
t (a2.306)		0.68	1.54	1.08

a Figures in parentheses are the theoretical t value at (*p*=0.05);

b Reference method: aliquots of standard solutions in methanol containing 2–10 μg/ml LNG were measured at 299 nm using methanol as a blank (2). No significant difference between groups by using one way ANOVA with F equals 0.78 and p equals 0.52.

## CONCLUSION

The proposed LC methods proved to be simple, accurate and reproducible for the determination of LNG in reasonable run times. The methods were validated showing satisfactory data for all the method validation parameters tested. The developed method can be conveniently used by quality control laboratories.
